# Tubulin is a molecular target of the Wnt-activating chemical probe

**DOI:** 10.1186/s12858-016-0066-9

**Published:** 2016-05-20

**Authors:** Yasunori Fukuda, Osamu Sano, Kenichi Kazetani, Koji Yamamoto, Hidehisa Iwata, Junji Matsui

**Affiliations:** Pharmaceutical Research Division, Takeda Pharmaceutical Company Limited, 2-26-1, Muraokahigashi, Fujisawa, Kanagawa Japan

## Abstract

**Background:**

In drug discovery research, cell-based phenotypic screening is an essential method for obtaining potential drug candidates. Revealing the mechanism of action is a key step on the path to drug discovery. However, elucidating the target molecules of hit compounds from phenotypic screening campaigns remains a difficult and troublesome process. Simple and efficient methods for identifying the target molecules are essential.

**Results:**

2-Amino-4-(3,4-(methylenedioxy)benzylamino)-6-(3-methoxyphenyl)pyrimidine (AMBMP) was identified as a senescence inducer from a phenotypic screening campaign. The compound is widely used as a Wnt agonist, although its target molecules remain to be clarified. To identify its target proteins, we compared a series of cellular assay results for the compound with our pathway profiling database. The database comprises the activities of compounds from simple assays of cellular reporter genes and cellular proliferations. In this database, compounds were classified on the basis of statistical analysis of their activities, which corresponded to a mechanism of action by the representative compounds. In addition, the mechanisms of action of the compounds of interest could be predicted using the database. Based on our database analysis, the compound was anticipated to be a tubulin disruptor, which was subsequently confirmed by its inhibitory activity of tubulin polymerization.

**Conclusion:**

These results demonstrate that tubulin is identified for the first time as a target molecule of the Wnt-activating small molecule and that this might have misled the conclusions of some previous studies. Moreover, the present study also emphasizes that our pathway profiling database is a simple and potent tool for revealing the mechanisms of action of hit compounds obtained from phenotypic screenings and off targets of chemical probes.

**Electronic supplementary material:**

The online version of this article (doi:10.1186/s12858-016-0066-9) contains supplementary material, which is available to authorized users.

## Background

Drug candidate selection through small-molecule screening is a rational and widespread method in the current drug discovery cascade. Initially, drug discovery research involved cell-based phenotypic screening as a core approach to obtaining drug candidates [1]. However, since the completion of the Human Genome Project in 2003 and the finding that sequences include numerous potential target proteins for drug discovery, target-based drug screening has been pursued actively [2, 3]. In addition, target-based drug screening procedures were initially accelerated to increase the research and development productivity of drug discovery in pharmaceutical companies. However, the number of FDA-approved drugs screened from the target-based approach was much less than expected because a large number of drug candidates failed during drug development owing to safety issues and a lack of efficacy [4]. In contrast, recent analysis of all first-in-class new molecular entities showed that phenotypic screening approaches accounted for 37 % in comparison with 23 % from target-based approaches [1]. Accordingly, classical cellular phenotypic screenings, also called phenotypic drug discovery (PDD), are being reevaluated as complementary and efficient strategies for probing drug candidates.

Chemical probes are powerful tools for target validation of hit compounds from PDD. However, some well-known chemical probes have been used incorrectly and have resulted in misleading biological conclusions [5]. Therefore, target identification of these compounds is essential for PDD. To date, target identification methods that use chemical proteomics or activity-based proteomics have been developed, and they have uncovered many unique target proteins associated with bioactive compounds [6, 7]. Although they are certainly useful methods, they require mass spectrometry instrumentation and further chemical syntheses to add tags to compounds of interest without deteriorating their activities. To determine the target molecules of compounds without affinity tags, Petrone et al. developed the chemical biological descriptor “high-throughput screening finger-print (HTS-FP)” that employs accumulated HTS data [8]. On the other hand, Frederick et al. developed a screening platform that consists of a series of reporter gene assays to disclose the mechanisms of action (MOAs) of compounds and by conducting assays in a quantitative HTS format [9, 10]. To develop a much simpler target identification approach with tag-free compounds, we exploited a pathway profiling database using only tens of cellular assays representing cellular signaling cascades through evaluation of compounds at a single concentration.

Oncology has become one of the largest therapeutic areas in the pharmaceutical industry. Various kinds of molecular targets and cellular signals have been reported to inhibit cancer growth. Among them, cellular senescence is considered to be the most important cellular phenotype for permanently arresting the cell cycle [11]. To date, reports have shown that genetic mutations and cellular stressors such as oxidative stress enhance cellular senescence and that some small molecules induce cellular senescence [12, 13]. In particular, compounds that induce cellular senescence are expected to be potent drugs for suppressing cancer growth [14]. Here we conducted a phenotypic screening campaign based on high-content cellular imaging to probe small molecules that induce cellular senescence.

## Results

### Pathway profiling database classifies compounds according to their MOA

The pathway profiling database mainly comprises reporter gene assays using firefly luciferase that cover 13 different signaling pathways and cellular proliferation assays with 7 commercially available cell lines (Table 1). These types of cellular assays are widely used in cell biology research and are highly accessible because of their simple procedures and low cost. In addition, these assays are very robust and demonstrate high throughput, which enabled us to detect subtle signal changes in an HTS-compatible format. The assays were functionally validated using the dose-dependent response of a natural ligand or known inhibitors/activators.

**Table 1:** Constituents of the pathway profiling database. The types of cellular signals for the reporter gene assays and cell lines of the proliferation assays are shown

Cellular reporter gene assays	Cellular proliferation assays
cAMP response element (CRE) signal	HEK293T
Nuclear factor of activated T-cells (NFAT) signal	MRC5 (high density)
Nuclear factor kappa- light-chain-enhancer of activated B cells (NF-kB) signal	MRC5 (low density)
Serum response element (SRE) signal	A549
Serum response factor (SRF) signal	PC3
p53 signal	LNCaP
E2F signal	Jurkat
Activating transcription factor 6 signal	MDA-MB-231
Hedgehog signal	
Hypoxia-inducible factor 1 (HIF1) signal	
Nuclear factor erythroid 2-related factor 2 (Nrf2) signal	
SMAD signal	
Wnt signal	

**Fig. 1:**
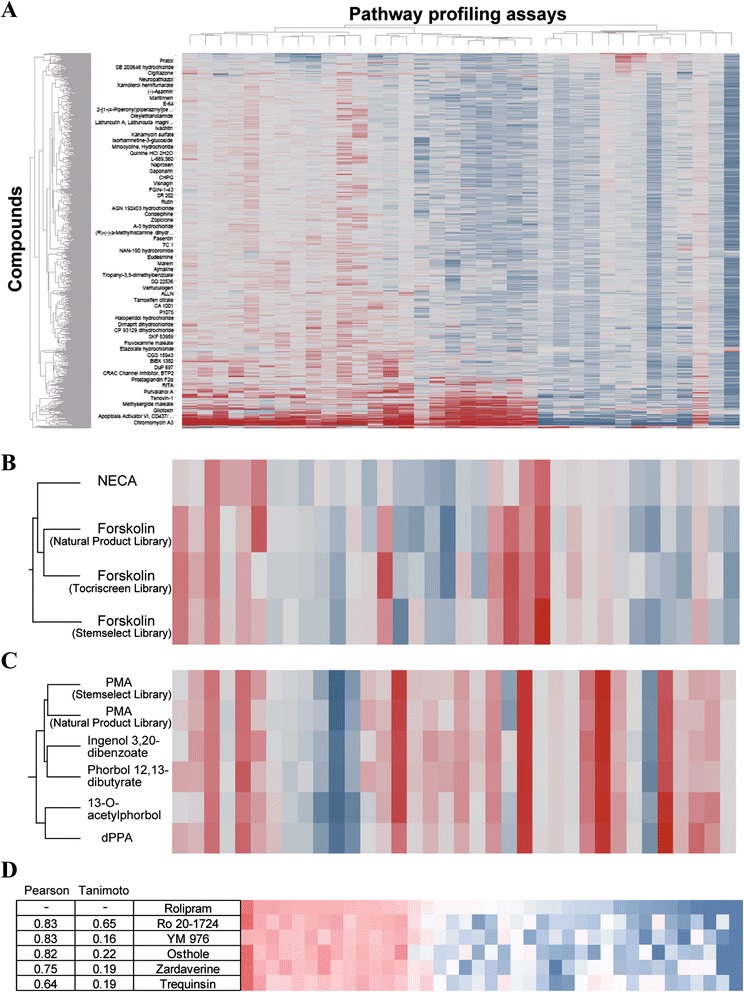
Analysis of the pathway profiling database. **a** The heat map was visualized with TIBCO Spotfire software for clustering analysis. This figure represents the entire heat map of the pathway profiling database. The activities of each assay are displayed as a gradient from minimum activities (blue) to maximum activities (red). For details of the assay lists, see Table 1. **b** This cluster contained forskolin derived from each commercial compound library and NECA, a potent adenosine receptor agonist. **c** PMA and its structural analogs were grouped in the cluster shown. **d** The Tanimoto structural similarities and Pearson’s correlation coefficients (activity versus activity) were calculated for PDE4 inhibitors

Through the development of this database, we evaluated 1910 compounds from 3 commercial compound libraries that contained compounds with well-characterized MOAs and common experimentally used reference compounds. We evaluated these libraries at a single concentration of 3 μg/mL for the Natural Product Library and at 3 μM for the other libraries. After obtaining all data, the database was analyzed using hierarchical clustering of the activities using Ward’s method in TIBCO Spotfire software (Fig. 1a). As a result of the hierarchical clustering analysis, compounds that had similar activities in most assays were classified into the same cluster, enabling us to visually determine that they have similar molecular targets and signaling pathways.

**Fig. 2:**
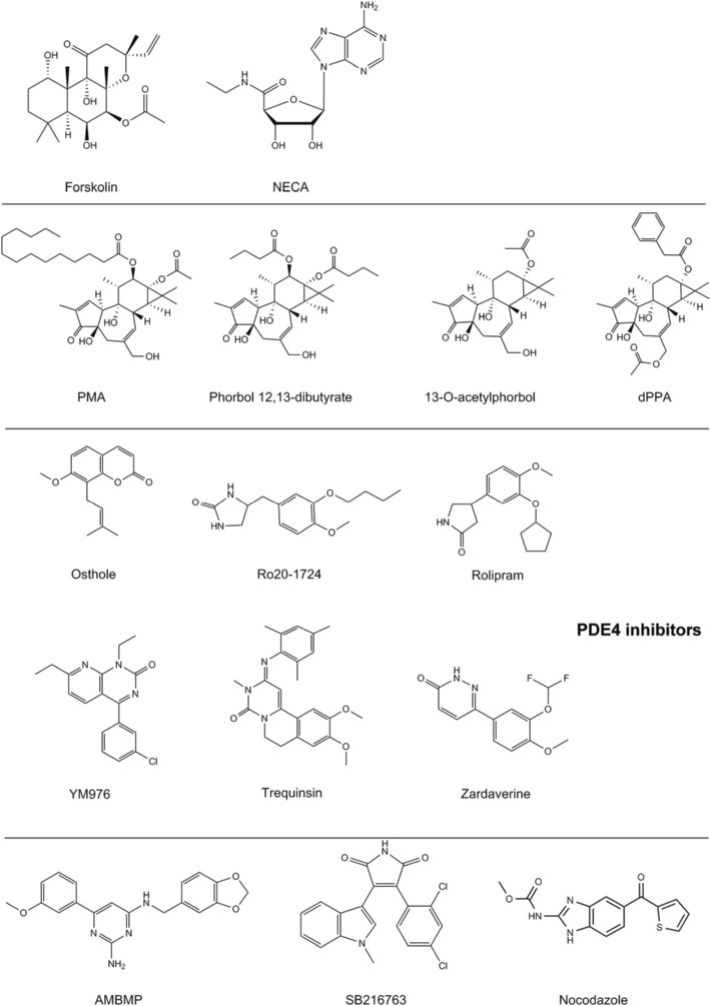
Chemical structures of the compounds discussed in this study

Forskolin (Fig. 2), an adenylate cyclase activator [15], was included in each library, and all were grouped into one cluster (Fig. 1b). In the cluster, N-ethylcarboxamidoadenosine (NECA) (Fig. 2), an adenosine receptor agonist [16], was also included. This cluster was shown to gather compounds stimulating cAMP production via adenylate cyclase activation. This result indicates that the pathway profiling database classifies compounds according to their MOA. Similarly, phorbol 12-myristate 13-acetate (PMA) [17] and its structural analogs phorbol 12,13-dibutyrate [18], 13-O-acetylphorbol [19], and 12-deoxyphorbol 13-phenylacetate 20-acetate (dPPA) [20] (Fig. 2) were classified into the same cluster (Fig. 1c). In other words, the structural analogs that had the same effect on cellular signaling were categorized into one cluster, as expected.

Following these analyses, we investigated structurally diverse compounds that affect the same target proteins. We focused on the phosphodiesterase (PDE) inhibitors [21–24] (Fig. 2) contained in our database. To quantitatively compare differences in the structures and activities of each compound in our database, we employed Tanimoto structural similarity calculated by Daylight’s fingerprints and Pearson’s correlation coefficients (activity versus activity), respectively. The Tanimoto similarities ranged from 0.16 to 0.65, strongly indicating the broad structural diversity between the compounds in our database (Fig. 1d). In contrast, Pearson’s correlation coefficients (activity versus activity) in our database ranged from 0.64 to 0.83 (Fig. 1d), showing their high bioactive similarities, despite their low structural similarities. These results indicate that our pathway profiling database based on the biological activities of compounds led to classifications corresponding to not only their structural similarities but also their MOAs.

### Wnt-activating small molecule is identified as a cellular senescence inducer

**Fig. 3:**
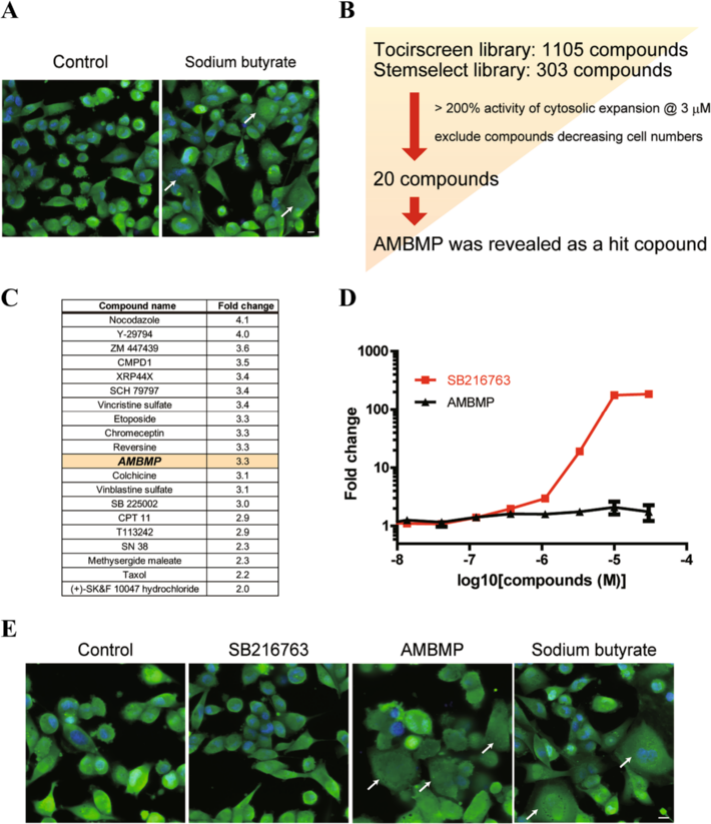
A cell-based assay for a screening campaign of cellular senescence morphology inducers by fluorescence microscopy. **a** MDA-MB231 cells were treated with 1 mM sodium butyrate. Hoechst 33342 was used as a nuclear marker (blue) and CMFDA was used to mark cytosols (green). Scale bar, 10 μm. **b** A compound selection scheme for the discovery of senescence inducers. AMBMP was obtained as a hit compound through the screening campaign. **c** Hit compound results from the screening campaign. Fold changes in the cellular area at 3 μM concentrations were calculated for the compounds with a custom-made image analysis algorithm. **d** Activity of the Wnt reporter gene assay with a potent GSK3β inhibitor, SB216763, and AMBMP is shown. The results are the mean of 3 replicate experiments (mean ± SD). **e** MDA-MB231 cells were treated with 3 μM SB216763, 3 μM AMBMP, and 1 mM sodium butyrate. Hoechst 33342 was used as a nuclear marker (blue) and CMFDA was used to mark cytosols (green). Scale bar, 10 μm

Triple-negative breast cancer has been a focus among the various cancer classes because of its lack of response to hormonal therapies, and new drugs with distinct MOAs are absolutely required to cure breast cancer patients [25]. Therefore, we employed MDA-MB-231 cells with triple-negative features to obtain cellular senescence inducers as anticancer agents [26]. In this strategy, we performed phenotypic screening on the basis of high-content cellular imaging, which is a very useful method to analyze altered cellular morphology. The cellular senescence morphology was reported to lead to a topologically enlarged appearance [11]. Sodium butyrate is a well-known senescence inducer [27], and we confirmed that it provoked the reported senescence phenotype in MDA-MB-231 cells and expanded cell shapes (Fig. 3a). In our study, this cellular morphology was defined as an indicator of cellular senescence.

For high-content screening (HCS) of senescence inducers, we developed a cell-based assay to analyze cellular phenotypic changes in MDA-MB-231 cells. To determine the activities of compounds in this HCS, the cellular area, which plays a key role in the selection of senescence inducers, was calculated using a custom-made image analysis algorithm. We screened 1408 compounds in Tocriscreen (TOCRIS Bioscience) and StemSelect Small Molecule Regulators (Merck Millipore) at concentrations of 3 μM and obtained 20 compounds that induced a ≥2-fold enlargement of the cytosolic area (Fig. 3b). Of these 20 compounds identified as senescence inducers (Fig. 3c), molecular targets of 19 compounds have been clarified in past studies, but that of 2-amino-4-(3,4-(methylenedioxy)benzylamino)-6-(3-methoxyphenyl)pyrimidine (AMBMP) (Fig. 2) has not been revealed yet. Thus, we focused on AMBMP to elucidate its molecular target, which is described further in this report.

It is generally considered that Wnt signaling pathways play important roles during embryonic development [28]. AMBMP was first identified as a Wnt signal agonist through Wnt signal activator screening using a common reporter gene assay [29]. To date, the first report of AMBMP has been cited in 68 papers, and the compound itself and its 10 applications have been patented (SciFinder®). However, its binding proteins have not yet been identified. We initially measured the activity of AMBMP using a Wnt reporter gene assay, as reported previously by Liu et al. [29]. Unexpectedly, using the Wnt reporter assay, we detected a much lower efficacy of AMBMP than that of a widely known Wnt signal activator glycogen synthase kinase 3β (GSK3β) inhibitor (SB216763) [30] (Figs. 2 and 3d). In contrast, GSK3β inhibitors were not observed to induce the senescence morphology (Fig. 3e, Additional file 1: Figure S1). These results strongly suggest that Wnt signal activation is not directly related to its cellular senescence and that AMBMP has binding proteins responsible for inducing cellular senescence.

### Pathway profiling database identifies tubulin as a target protein of AMBMP

**Fig. 4:**
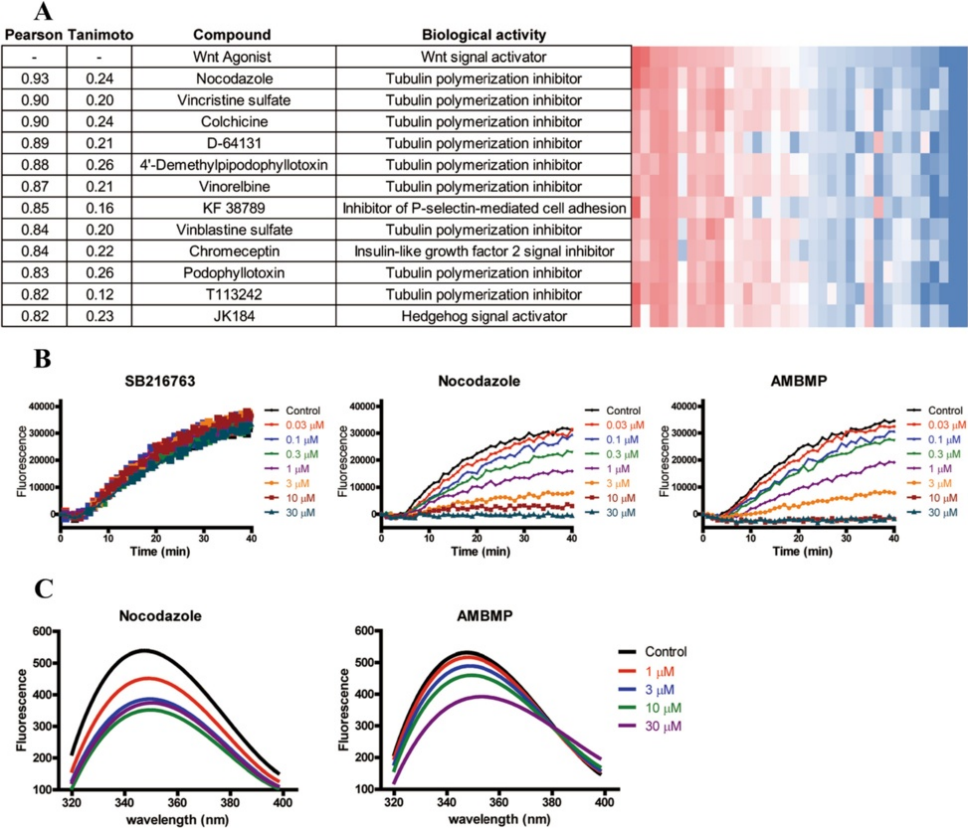
Target identification of AMBMP and its binding activity to tubulin. **a** The Tanimoto structural similarities and Pearson’s correlation coefficients (activity versus activity) were calculated against AMBMP. **b** SB216763, AMBMP, and nocodazole were evaluated in a tubulin polymerization assay. The results are the mean of 3 replicate experiments (with SD not shown for graphical simplicity). **c** AMBMP induced intrinsic tryptophan fluorescence spectra changes of tubulin. The results are the mean of 3 replicate experiments (with SD not shown for graphical simplicity)

To identify an AMBMP target molecule, we compared the cellular assays with our pathway profiling database and calculated each Pearson’s correlation coefficient (activity versus activity) between AMBMP and other all compounds in our database. As a result, 12 compounds demonstrated values above 0.8, which indicated high similarities (Fig. 4a). Moreover, 10 of the 12 compounds involved classical tubulin disruptors such as nocodazole (Fig. 2) and were thus known from previous reports to bind to tubulin [31–33]. Of these 10 compounds, only 2, KF 38789 and chromeceptin, had not been reported to induce tubulin depolymerization. The analyzed data allowed us to predict that AMBMP would directly interact with tubulin. To test this hypothesis, we measured the tubulin disruption activity of AMBMP in a tubulin polymerization assay. Consequently, tubulin polymerization was detected by fluorescence enhancement following uptake of a fluorescent reporter molecule into the polymerized tubulin during polymerization [34].

**Fig. 5:**
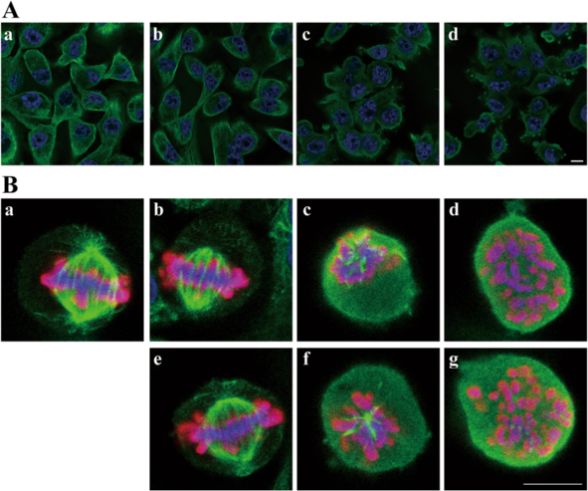
Effect of AMBMP on the cellular tubulin network and mitotic spindles. (A) The cellular tubulin network (green) was observed by fluorescence microscopy. Hoechst 33342 was used as a nuclear marker (blue). (a) control. (b) 3 μM SB216763. (c) 3 μM AMBMP. (d) 3 μM Nocodazole. Scale bar, 10 μm. (B) Control and compounds-treated MDA-MB-231 cells were stained with α-tubulin (green), phospho-histone H3 (red), and nuclei (blue). Phosphorylation at a highly conserved serine residue (Ser10) in the histone H3 is a key marker during the mitotic phase of the cell cycle. (a) control. (b) 30 nM nocodazole. (c) 0.3 μM nocodazole. (d) 3 μM nocodazole. (e) 30 nM AMBMP. (f) 0.3 μM AMBMP. (g) 3 μM AMBMP. Scale bar, 10 μm

We observed tubulin polymerization inhibition by AMBMP and nocodazole with IC_50_ values of 0.33 μM and 0.34 μM, respectively (Fig. 4b, Additional file 1: Figure S2A). In this fluorescence-based polymerization assay, AMBMP was confirmed not to mediate the fluorescence interference through the observation of its UV–vis and fluorescence spectrum (Additional file 1: Figure S3). In addition, intrinsic fluorescence quenching was used to study the potential interaction between AMBMP and tubulin. The fluorescence intensity of tubulin was decreased gradually with increasing concentrations of AMBMP, confirming its binding to tubulin. (Fig. 4c). To determine the effects of these 2 compounds on the cellular microtubule network, we conducted a cell-based assay using cellular imaging techniques and fluorescent staining of tubulin. In the confocal image analysis, AMBMP and nocodazole were observed to clearly disrupt the intracellular microtubule network compared to control and SB216763-treated cells (Fig. 5A). Disturbance of the microtubule network by AMBMP and nocodazole was detected with IC_50_ values of 0.34 μM and 1.7 μM, respectively (Additional file 1: Figure S2B). Furthermore, AMBMP as well as nocodazole was observed to inhibit cell proliferation and induce a cell cycle arrest in MDA-MB-231 cells (Additional file 1: Figure S4A, S4B). The effect of AMBMP on mitotic spindles was also observed with slightly shortening the spindle and astral microtubule at the low concentration of 30 nM and with significantly disrupting mitotic spindles at the higher concentrations of 0.3 and 3 μM (Fig. 5B), which was consistent with previous reports showing the effect of microtubule disruptors on mitotic spindles [35]. These results indicate that AMBMP had a strong inhibitory effect on tubulin polymerization, comparable to that of nocodazole. In addition, we had previously observed in our screening campaign that common tubulin disruptors induce cellular senescence (Fig. 3c) [36, 37].

## Discussion

In our study, the pathway profiling database based on the biological activities of compounds was confirmed to lead to classifications corresponding to both their structural similarities and their MOAs. Through operating the system, we will both maintain and obtain data at a lower cost and in a shorter period than the HTS-FP database and BioMAP™ (DiscoveRx), in which primary cells were utilized. However, our prediction method is limited to the range of target molecules of the reference compounds; however, to overcome this limitation, we will add various reference data for compounds that affect different types of target proteins other than those of the current compounds. In general, the accuracy of clustering analysis increases with a larger collection of datasets. Therefore, we will expand the cellular assays in the pathway profiling database to improve the accuracies of predicting both target molecules and cellular signaling properties. With these improvements in our system, we are attempting to perform target identification of other compounds, including our in-house compounds, with unknown targets.

In addition, we estimated the extent of cellular signaling pathways covered by our database through a computational approach. With Reactome Pathway Database [38], our pathway profiling database has the potential to detect cellular events involved in more than 200 canonical biological pathways. Moreover, 70 % of the tested compounds with well-characterized MOAs had detectable activity in at least one assay in our database. Consequently, our simple system is a promising and cost-effective tool for profiling phenotypes and for predicting molecular targets of hit compounds from PDD.

By applying our profiling system for target identification of AMBMP, we have revealed that AMBMP is a tubulin disrupting molecule for the first time since the compound was reported as a Wnt agonist. The Tanimoto similarities between AMBMP and tubulin disruptors ranged from 0.12 to 0.26 (Fig. 4a), which means that these compounds are apparently not structural analogs of AMBMP. Because of their low scores, the structural similarities did not lead us to hypothesize whether AMBMP could inhibit tubulin polymerization. The achievement of AMBMP target identification supports the result that our pathway profiling database was extremely useful for predicting various pharmacological targets of compounds with unknown mechanisms. On the other hand, we consider that it is important to reveal the molecular mechanisms inhibiting tubulin polymerization by AMBMP. To address the issue, in future study, we will clarify its binding site on tubulin through a cocrystal structural analysis for AMBMP and tubulin complex.

Chemical probes are widely used to demonstrate target molecule proof-of-concept in drug discovery [39]. To this end, the selectivity of chemical probes against the intended targets is a key factor. If these chemical probes interact with unintentional molecules and induce cellular phenotypes through their off-target effects, then both time and money might be lost in the process of drug discovery research. Some past research that used AMBMP as a chemical probe for Wnt signal activation might have incorrectly generated misleading results due to inhibition of tubulin activity. Recently, the met proto-oncogene (c-MET) inhibitor tivantinib was confirmed to inhibit tubulin polymerization as well as AMBMP [40]. Through our study, KF 38789 and chromeceptin were also shown to have similar bioactive profiles to tubulin disruptors (Fig. 4b), generating the possibility that both compounds interact with tubulin. These compounds will be the subject of a future publication. In addition, a previous report revealed that the structural similarities of compounds do not provide sufficient information to speculate on their biological activities [41]. For an efficient drug discovery process, it is important to evaluate and profile chemical probes using various types of cellular assays, such as our pathway profiling database.

## Conclusion

Our pathway profiling database determined tubulin to be a target of AMBMP, which was unknown since the discovery of AMBMP, and our simple and efficient system proved to be a powerful method for predicting compound MOAs. AMBMP has been widely used as a chemical probe for Wnt signal activation, but the results for studies that used the compound might have been influenced by its modulation of tubulin activity and not Wnt signal activity. For proper utilization of chemical probes, it is potentially valuable to investigate their cellular profiles using multiple cellular assays, such our pathway profiling database, which provides beneficial information about representative cellular signaling processes. Moreover, in drug discovery, off-target interactions are strongly thought to lead to low efficacy and significant side effects in clinical trials; therefore, the development of target identification and prediction methods is now definitively required to determine not only on-target molecules but also off-target molecules. The system will certainly keep providing us with useful information for various stages of the drug discovery process through target prediction and drug safety research.

## Methods

### Chemical compounds

Tocriscreen (TOCRIS Bioscience), Natural Product Library (ENZO Life Sciences), and StemSelect Small Molecule Regulators (Merck Millipore) were all dissolved in DMSO (10 mM for Tocriscreen and StemSelect and 10 mg/mL for the Natural Product Library). AMBMP was obtained from Merck Millipore. Sodium butyrate, nocodazole, and SB216763 were sourced from Wako.

### Cell cultures

HEK293T, MRC5, A549, PC3, LNCaP, Jurkat, MDA-MB231, NIH-3 T3, and SW480 cells were purchased from ATCC. HEK293T, A549, MRC5, and MDA-MB-231 cells were cultured in DMEM containing 4.5 g/L glucose, 10 % fetal bovine serum (FBS), and penicillin/streptomycin. Jurkat, LNCaP, SW480, and PC3 cells were cultured in RPMI 1640 media containing 10 % FBS and penicillin/streptomycin. NIIH-3 T3 cells were cultured in DMEM containing 1.5 g/L glucose, 10 % FBS, and penicillin/streptomycin. All cell culture reagents were purchased from Wako.

### Reporter gene assays in pathway profiling

We developed reporter gene assays using a firefly luciferase system purchased from Promega. Detailed assay conditions such as cell lines, cell densities, corresponding ligands, incubation time with compounds, and materials are shown (Table 2). All assays were performed in a 384-well plate format. Plasmids were constructed by inserting each response element sequence at a multi-cloning site upstream from firefly luciferase. Transient transfections of all plasmids were performed in corresponding cell lines with Fugene HD (Promega) according to the manufacturer’s instructions. In each assay, we validated the assay condition with its ligand to perform a stable screening campaign (data not shown). In all assays, all compounds were diluted in complete media at a concentration of 3 μg/mL (Natural Product Library) and 3 μM (other libraries) and treated for the appropriate durations. After the addition of Steady-Glo (Promega) according to the manufacturer’s instructions, luminescence signals were measured using a luminescence plate reader (EnVision; PerkinElmer). We typically obtained 2 parameters calculated from each assay: one was the compound’s inhibitory activity with ligand activation and the other was its agonistic activity without ligand activation.

**Table 2:** Assay conditions such as cell lines, cell densities, corresponding ligands, incubation time with compounds, and materials for reporter gene assays in pathway profiling

Cellular signal	Cell lines	Cell density (cells/well)	Ligand	Incubation time with compounds	Original materials or references
cAMP response element (CRE) signal	HEK293T	5,000	Forskolin (1 μM)	5 h	pGL4.29 (Promega)
Nuclear factor of activated T-cells (NFAT) signal	HEK293T	5,000	Ionomycin (1 μM) PMA (10 ng/mL)	5 h	pGL4.30 (Promega)
Nuclear factor kappa-light-chain-enhancer of activated B cells (NF-kB) signal	HEK293T	10,000	TNFα (3 ng/mL)	20 h	pGL4.32 (Promega)
Serum response element (SRE) signal	HEK293T	20,000	FBS (15 %) PMA (30 ng/mL)	20 h	pGL4.33 (Promega)
Serum response factor (SRF) signal	HEK293T	20,000	FBS (15 %)	5 h	pGL4.34 (Promega)
p53 signal	HEK293T	10,000	Doxorubicin (1 μM)	20 h	[43]
E2F signal	HEK293T	20,000	FBS (15 %)	20 h	[44]
Activating transcription factor 6 signal	HEK293T	5,000	Thapsigargin (30 nM)	5 h	[45]
Hedgehog signal	NIH3T3	7,500	mouse sonic hedgehog	20 h	[46]
Hypoxia-inducible factor 1 (HIF1) signal	HEK293T	10,000	hypoxia	20 h	[47]
Nuclear factor erythroid 2-related factor 2 (Nrf2) signal	HEK293T	5,000	tert-butylhydroquinone (20 μM)	20 h	pGL4.37 (Promega)
SMAD signal	HEK293T	10,000	TGFβ (0.2 ng/mL)	20 h	[48]
Wnt signal	HEK293T	10,000	Wnt3a	20 h	[49]
Wnt signal	SW480	10,000	no ligand (constitutive active)	20 h	[49]
IL17 signal	Jurkat	5,000	Ionomycin (400 nM) PMA (4 ng/mL)	5 h	[50]

### Cellular proliferation assays used in pathway profiling

Cell lines, cell densities, and incubation times with compounds are shown (Table 3). Cellular proliferation was detected with CellTiter-Glo (Promega). All assays were performed in a 384-well plate format. Luminescence signals were readout using a luminescence plate reader (EnVision; PerkinElmer). The proliferation assays with HEK293T cells and Jurkat cells were used as the counter-screen against reporter gene assays.

**Table 3:** Assay conditions such as cell lines, cell densities, and incubation times for cellular proliferation assays in pathway profiling

Cell lines	Cell density (cells/well)	Incubation time with compounds
HEK293T	5,000	20 h
Jurkat	5,000	20 h
MRC5	1,000	72 h
MRC5	3,500	72 h
A549	1,000	72 h
PC3	1,000	72 h
LNCaP	600	72 h
MDA-MB-231	500	72 h

### Cell-based phenotypic assays for cellular senescence inducers

MDA-MB231 cells were seeded in a 384-well plate (3000 cells/well) for 20 h before the treatment of compounds. After seeding, the tested compounds were diluted in complete media and incubated with cells for 24 h, followed by cytosol and nuclear staining for 1 h with CellTracker Green CMFDA and Hoechst 33342 (Invitrogen), respectively. For cellular tubulin staining, tubulin tracker green was used according to the manufacturer’s instructions (Invitrogen). Cellular images were recorded with an IN Cell Analyzer 6000 (GE Healthcare). After obtaining the images, the nuclear locations and cellular areas were stained with Hoechst 33342 and CMFDA, respectively, and quantitative signals from the images were calculated using a custom-made image analysis algorithm with IN Cell Developer Toolbox (GE Healthcare).

### Cluster analysis in the pathway profiling system

All compounds were utilized at a concentration of 3 μg/mL (Natural Product Library) or 3 μM (other libraries) in the pathway profiling assays. All calculated data, including percent inhibition and percent activation number, were first normalized in each assay using the Z-scoring method and then analyzed by hierarchical clustering analysis (Ward’s method) with TIBCO Spotfire software (TIBCO).

### Calculating Pearson’s correlation coefficients

Pearson’s correlation coefficients (Rp) were calculated using the following equation:

$$ \mathrm{R}\mathrm{p} = \frac{{\displaystyle {\sum}_{i=1}^N}\left({x}_i-\overline{x}\right)\left({y}_i-\overline{y}\right)}{\sqrt{{\displaystyle {\sum}_{i=1}^N}{\left({x}_i-\overline{x}\right)}^2}\sqrt{{\displaystyle {\sum}_{i=1}^N}{\left({y}_i-\overline{y}\right)}^2}} $$

where N equals 39 assay results and *x*_i_ and *y*_i_ are the activity values of each assay in our pathway profiling database for compounds A and B, respectively.

### Tubulin polymerization assay

Tubulin polymerization was performed using a tubulin polymerization assay kit (BK011P, Cytoskeleton). Compounds were evaluated according to the manufacturer’s instructions.

### Tubulin binding assay with its intrinsic tryptophan fluorescence

4 μM of purified tubulin (Cytoskeleton) dissolved in general tubulin buffer (80 mM PIPES, pH 6.9, 2 mM MgCl_2_, 0.5 mM EGTA) was pretreated with certain concentrations of compounds for 30 min. The intrinsic fluorescence spectra (320–400 nm) was measured with a fluorescence plate reader (EnVision; PerkinElmer) with the excitation wavelength 295 nm.

### Immunofluorescence microscopy

MDA-MB231 cells were incubated with compounds for 6 h and 24 h to observe the cellular microtubule network and the mitotic spindles respectively. Thereafter, the cells were fixed and permeabilized as described in the past report [42]. After blocking nonspecific binding with 1 % donkey serum/PBS, the cells were incubated with the mouse monoclonal anti-α-tubulin antibody (Cell Signaling Technology) (1:1000 dilution) followed by the Alexa-488 conjugated anti-mouse IgG antibody (Invitrogen) (1:500 dilution). To visualized nuclei, the cells were incubated with Hoechst33342. For staining phospho-Histone H3, the fixed cells were treated with the rabbit monoclonal anti-phospho-Histone H3 (Ser10) antibody (Cell Signaling Technology) (1:1000 dilution) followed by the Alexa-594 conjugated anti rabbit IgG antibody (Invitrogen) (1:500 dilution). Cellular images were obtained with SP8 confocal microscopy (Leica).

### Flow cytometric analysis

MDA-MB-231 cells were treated with compounds for 24 h, and fixed with ethanol. After fixation, cells were washed with PBS containing 2 % FCS, and, subsequently, treated with Guava Cell Cycle reagent (Merck Millipore) according to the manufacturer's instructions. The DNA contents were determined using a Guava easyCyte HT software (Merck Millipore).

## Additional file

Additional file 1: Supplemental results of cell-based assays and tubulin polymerization assay. **Figure S1**. Measurements of fold changes in the cellular area. **Figure S2**. Inhibitory activity on the tubulin polymerization and the cellular microtubule network. **Figure S3**. The absorbance and fluorescence profiles of AMBMP. **Figure S4**. Measurements of growth inhibitory activity, cell cycle distribution, and mitotic spindle of MDA-MB231 cells treated with AMBMP. (DOCX 6384 kb)

## Abbreviations

FDA: The Food and Drug Administration
HTS: high-throughput screening
