# Supplementary material for: Tubulin is a molecular target of the Wnt-activating chemical probe
Source: BMC Biochem. 2016 May 20;17:9. doi: 10.1186/s12858-016-0066-9 (PMC4873989; doi:10.1186/s12858-016-0066-9)
Supplement: Additional file 1: — Supplemental results of cell-based assays and tubulin polymerization assay. Figure S1. Measurements of fold changes in the cellular area. Figure S2. Inhibitory activity on the tubulin polymerization and the cellular microtubule network. Figure S3. The absorbance and fluorescence profiles of AMBMP. Figure S4. Measurements of growth inhibitory activity, cell cycle distribution, and mitotic spindle of MDA-MB231 cells treated with AMBMP. (DOCX 6384 kb) [file 12858_2016_66_MOESM1_ESM.docx]

**Supporting Information**

**Supplemental results of cell-based assays and tubulin polymerization assay**

**Figure S1.** Measurements of fold changes in the cellular area

MDA-MB231 cells were treated with 3 μM SB216763, 3 μM AMBMP, and 1 mM sodium butyrate. To measure the cellular area, cell cytosol was stained with CMFDA and detected by IN Cell Analyzer 6000 (GE Healthcare). Fold changes in the cellular area were calculated using a custom-made image analysis algorithm with IN Cell Developer Toolbox (GE Healthcare). The results are the mean of 2 replicate experiments (means ± SD).

**Figure S1.**


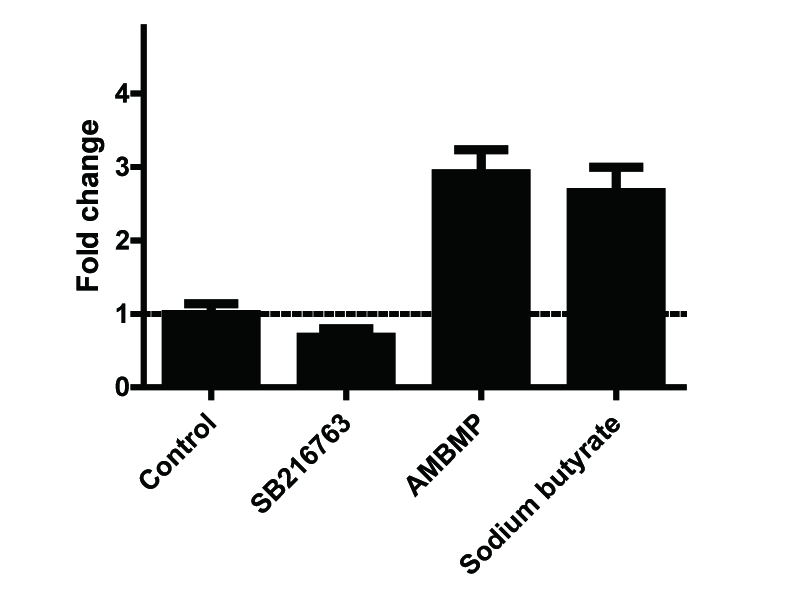


**Figure S2.** Inhibitory activity on the tubulin polymerization and the cellular microtubule network

**(A)** IC_50_ values of SB216763, AMBMP, and nocodazole were determined in a tubulin polymerization assay. The values were calculated using GraphPad Prism (GraphPad Software). **(B)** The fluorescence intensities of tubulin stained with a tubulin tracker (Invitrogen) were measured by IN Cell Analyzer 6000. IC_50_ values of SB216763, AMBMP, and nocodazole were determined using a custom-made image analysis algorithm with IN Cell Developer Toolbox. The values were calculated by GraphPad Prism. Both results are the mean of 3 replicate experiments (mean± SD).

**Figure S2.**


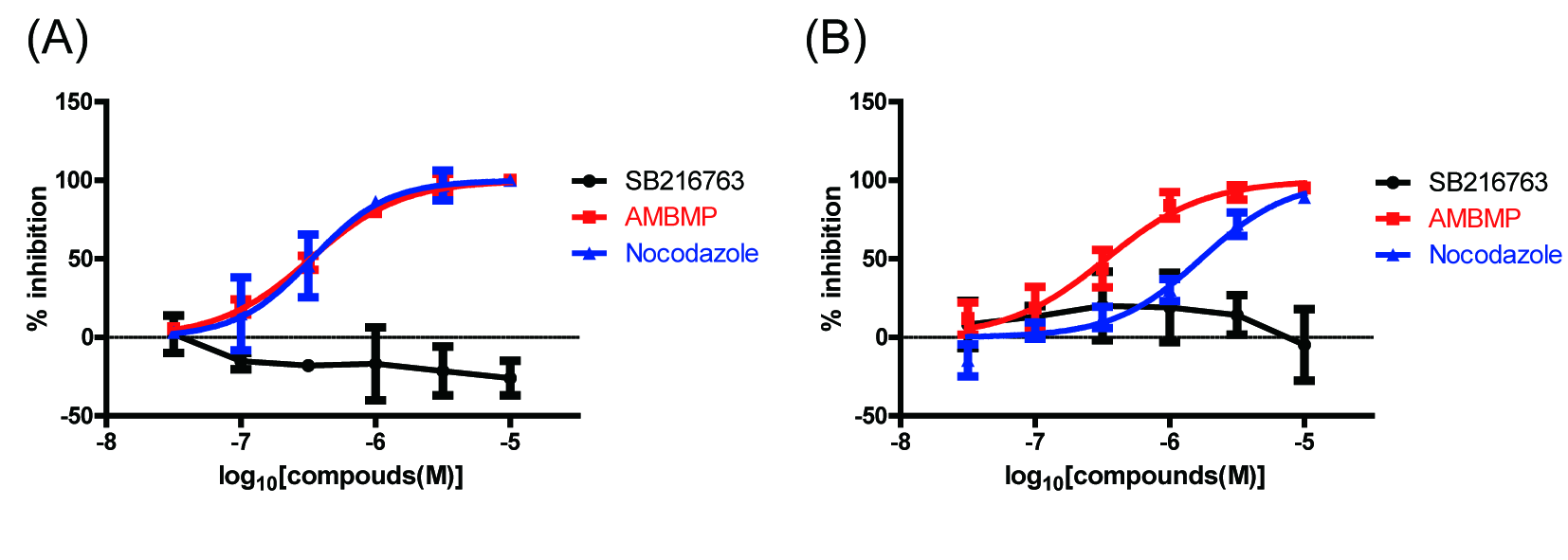


**Figure S3.** The absorbance and fluorescence profiles of AMBMP.

**(A)** UV-Vis spectra of AMBMP were observed with NanoDrop 1000 (Thermo Fisher Scientific).

**(B)** Emission spectra of AMBMP. The excitation wavelength was 350 nm and emission spectra were acquired by scanning from 380 to 500 nm using EnVision (Perkinelmer).

**(A)**

**(B)**

**Figure S4.** Measurements of growth inhibitory activity, cell cycle distribution, and mitotic spindle of MDA-MB231 cells treated with AMBMP

**(A)** The growth inhibitory activity of AMBMP and nocodazole were detected in a cell proliferation assay with the half-maximal inhibition of proliferation (IC_50_) values of 58 nM and 43 nM, respectively. The values were calculated using GraphPad Prism (GraphPad Software). The results are the mean of 3 replicate experiments (means ± SD).

**(B)** MDA-MB-231 cells were treated with DMSO control, 3 μM AMBMP, 3 μM nocodazole. The cellular DNA contents were determined with flow cytometric analysis. The results are the mean of 4 replicate experiments (means ± SD).

**(A)**


**(B)**


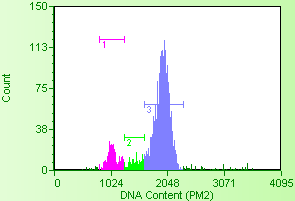

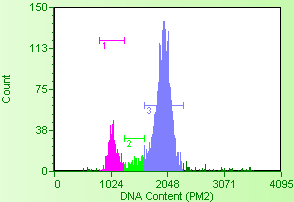

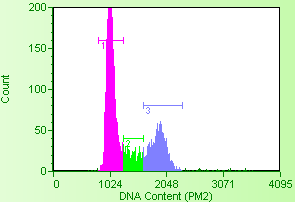


Control

AMBMP

Nocodazole
